# 188. Outcomes After Implementing the OPTIONS-DC Multidisciplinary Care Conference for Patients with Substance Use Disorders and Severe Bacterial Infections

**DOI:** 10.1093/ofid/ofab466.390

**Published:** 2021-12-04

**Authors:** Brenton J Schneider, Amber C Streifel, Cara D Varley, Michael Conte, Monica K Sikka

**Affiliations:** 1 OHSU, Portland, Oregon; 2 Oregon Health & Science University, Portland, OR

## Abstract

**Background:**

Hospitalizations for patients with severe bacterial infections (SBI) and substance use disorders (SUDs) are increasing. To address the unique treatment challenges for these patients and balance appropriate medical therapy with patient goals, we implemented the OPTIONS-DC, a structured multidisciplinary discharge planning conference. All patients with SBI and SUD at our institution qualify for an OPTIONS-DC.

**Methods:**

We performed a retrospective case-control study to evaluate differences and describe outcomes in patients who received an OPTIONS-DC compared to those who did not. Admissions were included if the patient was diagnosed with a SUD, a SBI requiring at least 2 weeks of antibiotics, consultation by infectious diseases and addiction medicine, and who were admitted between February 2018 and March 2020 (following implementation of OPTIONS-DC conferences). Patients were excluded for infected prosthetic material, pregnancy, or non-bacterial infection.

**Results:**

173 admissions qualified for inclusion and 73 had at least one OPTIONS-DC. Unstable housing and psychiatric disease were common (table 1). Opioid and methamphetamine use disorders were most common and almost all SUDs were severe. Patients who received an OPTIONS-DC had less medical comorbidities, less unstable housing, and were more likely to have an opioid use disorder, use more than one substance, start MAT while inpatient, and have vertebral osteomyelitis or epidural abscess (table 2). Patients who had a conference had similar proportions of unexpected discharges (13.7% vs 17%), but a higher proportion of treatment completion (83.6% vs 69%), more days of antibiotic therapy remaining after discharge (13.9 vs 9.8 days), were more likely to discharge to an outpatient setting with family or medical support (30% vs 9%), and more likely to complete their antibiotic course with a long-acting injectable (27.4% vs 9%)(table 3).

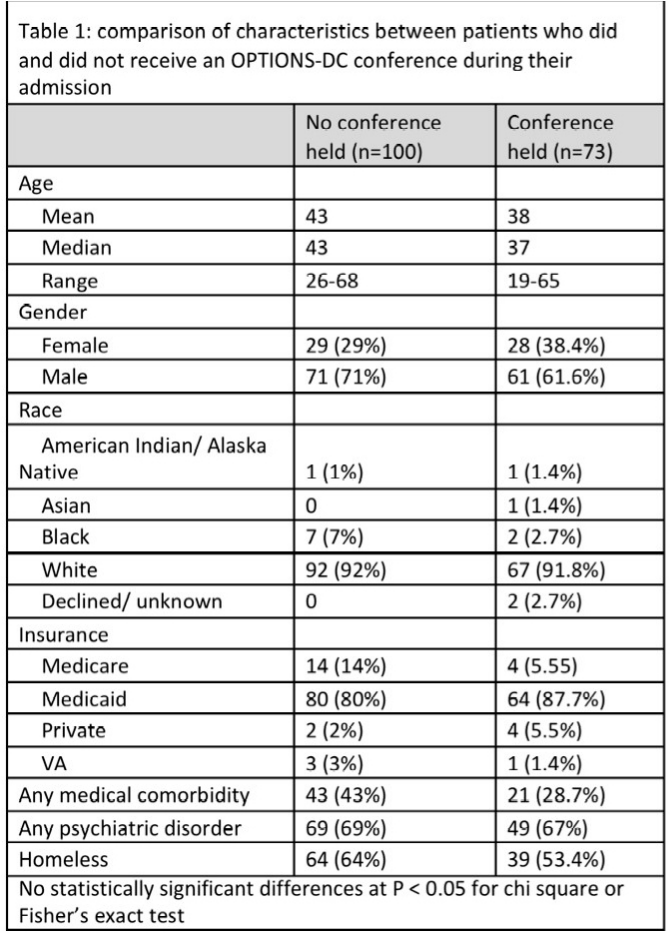

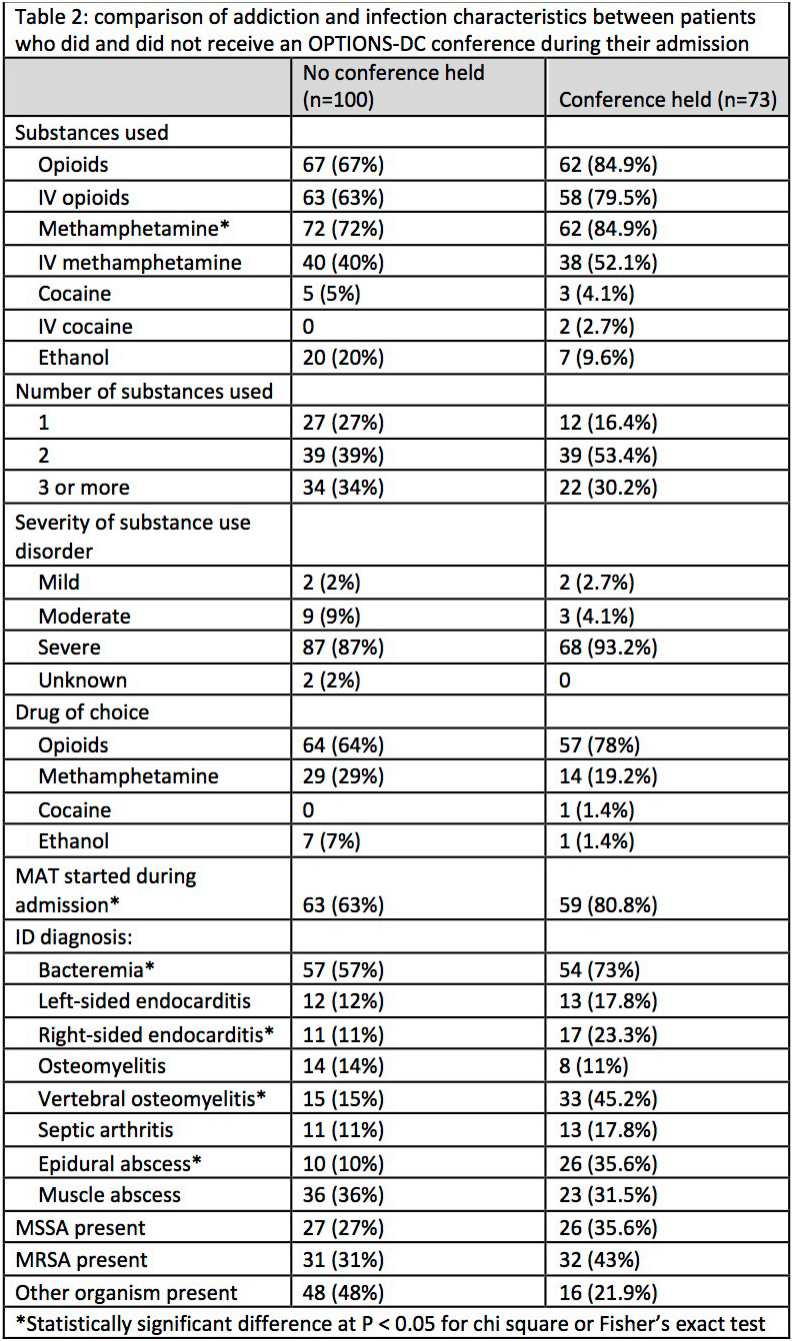

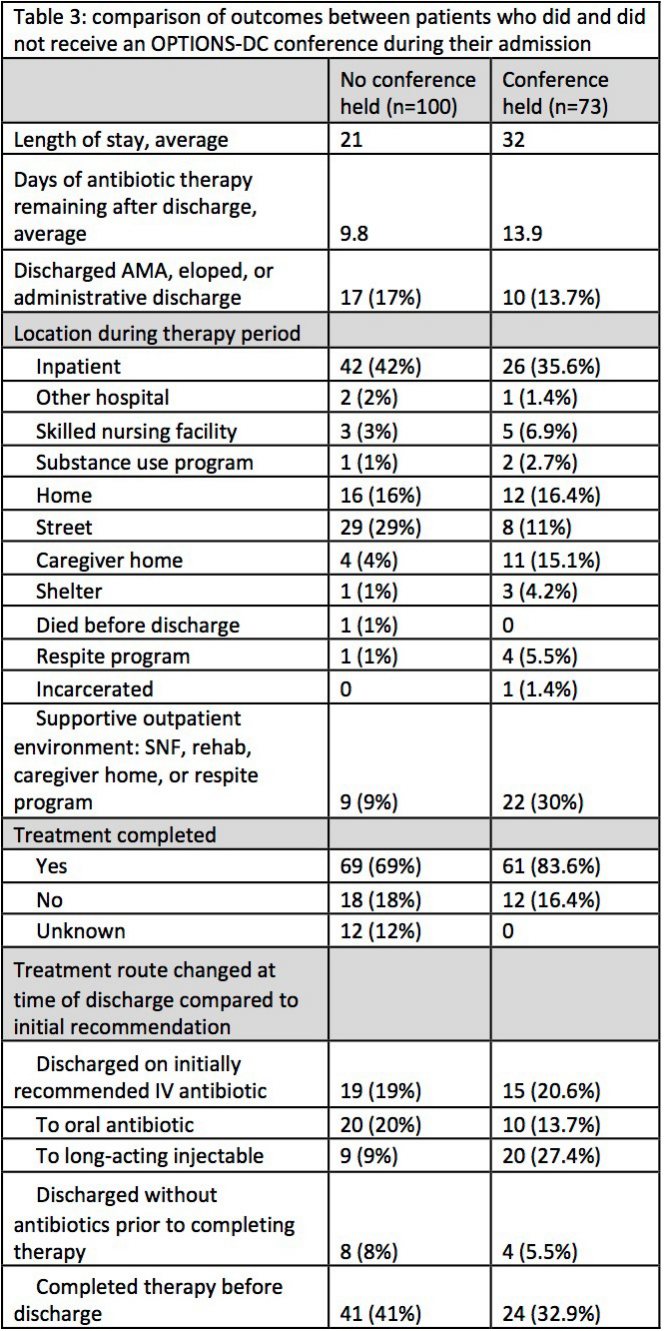

**Conclusion:**

Not all eligible patients received an OPTIONS-DC and there were significant differences in substances used, housing status and type of infections between those groups. Descriptive data suggest that OPTIONS-DC may reduce the duration of inpatient antibiotic treatment and increase likelihood of completion of antibiotic therapy, however this requires further study.

**Disclosures:**

**Amber C. Streifel, PharmD, BCPS**, **Melinta** (Advisor or Review Panel member) **Monica K. Sikka, MD**, **FG2** (Scientific Research Study Investigator)

